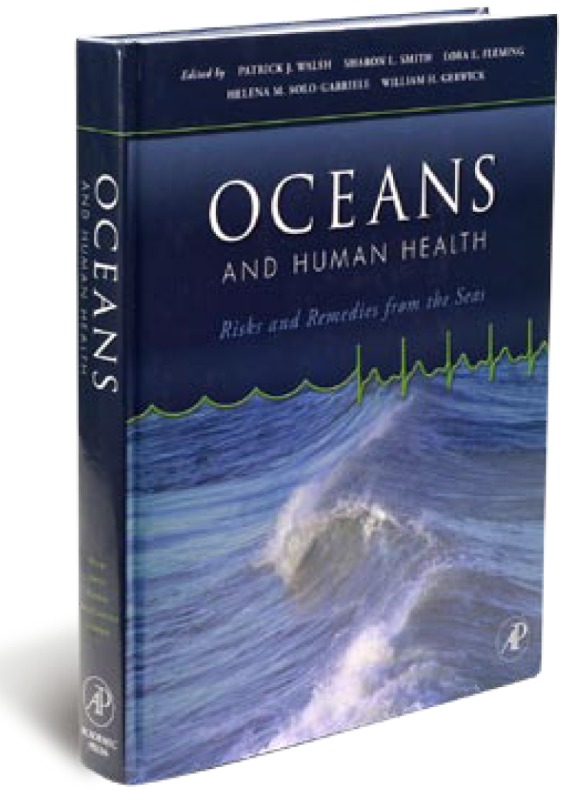# Oceans and Human Health: Risks and Remedies from the Sea

**Published:** 2009-03

**Authors:** D. Jay Grimes

**Affiliations:** D. Jay Grimes is professor of coastal sciences at the University of Southern Mississippi. He currently chairs the NOAA National Advisory Panel on Oceans and Human Health, is a science advisor to the Joint Ocean Commission Initiative, and is the co-vice chair of the Gordon Research Conference on Oceans and Human Health. Most recently, Grimes has focused his research on the distribution of and human health risks from waterborne pathogens, especially *Vibrio* species.

The editors of *Oceans and Human Health* have produced a very useful and authoritative resource book on a new (or at least consolidated) field of study: the relationships between the oceans and human health. Five oceans and human health (OHH) practitioners have teamed with an additional 87 authors to create this unique reference book.

OHH is an emerging “meta-discipline” that unifies previously unrelated disciplines including: oceanography, waterborne and seafood-borne diseases, harmful and nuisance algal blooms, epidemiology, comparative animal physiology, natural products and synthetic organic chemistry, pharmacology, toxicology, social sciences, engineering, natural disasters, and other related areas. The authors have broad and significant experience in the various aspects of this meta-discipline, and they provide thorough albeit not always uniform coverage of their fields. The book’s 33 chapters are arranged into two broad sections: “Risks” and “Remedies.” “Risks” covers various aspects of natural disasters, chemical pollution, harmful algal blooms, and waterborne and seafood-borne infectious diseases. “Remedies” focuses on pharmaceuticals and other natural marine products and on aquatic animals as models of human health. Each chapter contains a useful (and usually extensive) reference section; concluding each chapter are study questions designed to facilitate student comprehension. The book also contains case studies that “illustrate and reinforce the concepts covered.” Although case studies are indeed useful, there are only 10 such illustrative studies and they are limited to “Risks.” A second edition of the book should provide a glossary to the case studies by page and topic, and the other authors should be encouraged to provide case studies (e.g., results of structure assignments, preclinical evaluation, clinical trials, sustainable production). Finally, *Oceans and Human Health* presents topics in a logical manner; it is easy to read and it contains ample (there could be more) tables and figures that nicely illustrate concepts and facts described in the narrative. In general, the book is current, as evidenced by citations to recent publications. Students appreciate and use a highly detailed textbook, often referring to it years after the course has been completed. This book certainly provides long-term value for students.

Several of our graduate students and one of my co-instructors responded to my request for comments on the book, and they provided some useful input: “The book was very informative and an easy read.” “Texts usually focus on ecological issues. However, this text includes a good bit of information that is normally acquired in the field such as monitoring, equipment, and molecular techniques. … the coverage of global warming could have been more balanced and presented scientific evidence from both sides of the debate.” “I love the book. I enjoyed reading it. … It gives accessible reviews on a variety of disciplines that I found complimentary to my personal background.” “For young scientists especially, it gives many thought-provoking jumping-off points for future research.” And “The hurricane chapter was U.S.-centric. It would have been interesting to include a chapter or table(s) that specifically compared/listed the number and severity of cases from each OHH issue (hurricanes, each bacterium, viruses, etc.) over a period of time.” In general, the students enjoyed the book and found it useful.

Some specific problems include lack of reference to integrated ocean observing systems in Chapter 20; absence of a case study catalog or index; lack of a consistent style for presentation, including chemical structure; redundancy across some of the chapters (difficult to eliminate with so many different authors preparing the various chapters); limited coverage of socioeconomics; and too much coverage (more than 100 pages) given to aquatic animal models in “Remedies.”

Overall, I liked the book, and I will use it again as a textbook. It is also a useful reference book for both students and professionals new to this meta-discipline. The editors assembled an outstanding group of authors to write the chapters, and they have nicely covered every aspect of OHH. I feel fortunate to have this book in my personal library and sincerely hope that the editors compile future editions.

## Figures and Tables

**Figure:**